# Affective responses to uncertain real-world outcomes: Sentiment change on Twitter

**DOI:** 10.1371/journal.pone.0212489

**Published:** 2019-02-27

**Authors:** Sudeep Bhatia, Barbara Mellers, Lukasz Walasek

**Affiliations:** 1 Department of Psychology, University of Pennsylvania, Philadelphia, PA, United States of America; 2 WMG, University of Warwick, Coventry, West Midlands, United Kingdom; Central University of Finanace and Economics, CHINA

## Abstract

We use data from Twitter.com to study the interplay between affect and expectations about uncertain outcomes. In two studies, we obtained tweets about candidates in the 2014 US Senate elections and tweets about National Football League (NFL) teams in the 2014/2015 NFL season. We chose these events because a) their outcomes are highly uncertain and b) they attract a lot of attention and feature heavily in the communication on social media. We also obtained a priori expectations for the events from political forecasting and sport betting websites. Using this quasi-experimental design, we found that unexpected events are associated with more intense affect than expected events. Moreover, the effect of expectations is larger for outcomes that fall below expectations than outcomes that exceed expectations. Our results are consistent with fundamental principles in psychological science, such as reference-dependence in experienced affect. We discuss how naturally occurring online data can be used to test psychological predictions and develop novel psychological insights.

## Introduction

How do we feel when a favored political candidate loses an election or a home team beats last year’s champion? A key determinant of our affective responses is our beliefs about the event, with unexpected events leading to more intense affective responses than expected events [1–3]. When evaluating an event, we compare the outcome of the event to a reference point. Often, the reference point is the expected outcome. When the actual outcome exceeds the expectation, it is evaluated even more positively. When it falls short of the expectation, it is evaluated negatively.

Theoretical frameworks in which affective responses depend on reference points are abundant in psychology, economics, and political science. These frameworks include theories of motivation and causal attribution in social psychology [4,5], reinforcement learning models in cognitive psychology and neuroscience [6–8], theories of preference in judgment and decision-making and behavioral economics [9–13], and aspiration based models in politics [14]. These models share the assumption that evaluative processes involve expectations about outcomes, and they often assume that the psychological impact of losses and gains is not the same (also see [15]). Taken together, a shared principle that we focus on in the present paper is that expectations influence affect, and the interaction between outcomes and beliefs influences emotional reactions to a wide range of human behavior.

Although the psychological principles of reference-dependence have been extensively studied, much of the existing evidence is confined to the controlled laboratory settings, where outcomes are artificial or hypothetical. This is understandable. It is often difficult to quantify the evaluations of outcomes in real-world settings. Additionally, the precision necessary to study the interactions between expectation and affect can best be achieved by experimental manipulation. A dataset that captures these two variables in a naturalistic setting is, however, very valuable. First, large volumes of unsolicited data are not constrained by the features of the experimental designs, and may therefore provide more conservative tests of existing theories. Second, naturalistic data can offer novel insights when it includes information that is not easily manipulated/measured in the experimental setting (e.g., time course, geographical location). Consequently, if the dataset is rich enough in contextual information, it can be used to examine variables that moderate the predictions of theories.

In the following paper, we present a novel approach to study the interplay between affect and expectations in a naturalistic setting. We present the results of two studies analyzing millions of online posts (tweets) from the social networking site, Twitter. In particular, we obtained tweets about candidates in the 2014 US Senate elections and tweets about National Football League (NFL) teams in the 2014/2015 NFL season. We also obtained a priori expectations for these elections and games using predictions of political forecasters and point spreads offered by popular betting websites. Our goal was to examine whether the affective content of the tweets was influenced by the expected outcomes of the elections and games. More specifically, we assessed how tweets’ affect (as measured by their sentiment score) changed when the results of Senate elections and NFL matches became known. Our prediction was that the expectations people held about these outcomes would influence the strength of sentiment change. Both surprising losses and surprising wins should produce stronger affective responses than losses and wins that were unsurprising. As such, we set out to determine whether the results of our quasi-experiments adhere to affective and cognitive theories of psychology.

## Study 1: United States Senate elections

### Materials and methods

Study 1 examined tweet affect in the United States Senate elections held on Tuesday, November 4, 2014. We chose United States Senate election for two reasons. First, Senate elections are prolific enough to feature heavily in the communication on social media. We were therefore able to obtain large volumes of tweets relating to elections in different geographical regions (US States). Second, while outcomes of the elections are highly uncertain, it is possible to obtain predictions for them from various forecasting sites. On the day before the elections, we obtained predictions from five popular political forecasting newsletters and blogs: The Cook Political Report, Sabato’s Crystal Ball, The Rothenberg Political Report, Real Clear Politics, and Daily Kos Elections. Predictions for each of these blogs were made on a scale with labels of Safe Democrat, Likely Democrat, Lean Democrat, Tossup/Tilt Democrat, Tossup, Tossup/Tilt Republican, Lean Republican, Likely Republican, and Safe Republican. We averaged predictions over blogs to generate an aggregate expectation for each election, ranging from -4 (Safe Republican) to +4 (Safe Democrat). We then recoded predictions into a variable that captured the *expected outcome* for each candidate. This variable, based on whether the winning candidate would be a Democrat or Republican, ranged from -4 (very likely to lose) to +4 (very likely to win).

We also obtained 372,981 tweets about the candidates in these elections, posted in a two-week period between October 31^st^ and November 14^th^. Using Twitter’s data streaming feature (official Twitter API), we collected all tweets that mentioned the full names of the Democratic and Republican candidates for 19 Senate seats that were not considered safely Democrat or safely Republican by all five of our blogs (i.e. seats with expected outcomes that were strictly greater than -4 and strictly less than +4). We excluded these cases as we were mainly interested in events that were most uncertain–i.e. where the expectations are most likely to not match reality. We removed tweets mentioning two or more candidates and removed tweets about the Louisiana and Alaska elections because Louisiana has a “jungle” primary system in which runoff elections are held if no candidate receives a majority of votes. Indeed, in the 2014 elections this was thee case, and the winner was not determined until December, 2014. The 2014 election in Alaska was exceptionally tight, and final results were not confirmed until November 17, 2014. All other Senate races were resolved by the end of Election Day on November 4, 2014. Note that our study is purely observational, in compliance with Twitter user agreements, and the tweets were analyzed without any threat to personal privacy.

To calculate *tweet affect w*e used the Sentiment140 lexicon [16], combined with pointwise mutual information methods (PMI) [17]. This popular lexicon is based on a corpus of 1.6 million preexisting tweets with positively and negatively valenced *emoticons*. PMI methods assign to each of the 62,468 unique words in the lexicon an affective rating between -5 and +5 based on their co-occurrence with positively and negatively valenced emoticons. Words that commonly occur with positive emoticons, but not with negative emoticons, are considered high in positive affect. Similarly, words that commonly occur with negative emoticons, but not with positive ones, are considered high in negative affect. More formally, for items *x* and *y* (e.g. the word “happy” and the “smiley face” emoticon), the pointwise mutual information of the two items is given by $PMI(x,y)=log\left(\frac{p(x,y)}{p(x)p(y)}\right)$. Here *p*(*x*,*y*) is the probability of the co-occurrence of *x* and *y* in the corpus, and *p*(*x*) and *p*(*y*) are the individual probabilities of occurrence of *x* and *y* in the corpus. Controlling for *p*(*x*) and *p*(*y*), the PMI of *x* and *y* will be larger if *p*(*x*,*y*) is larger, that is, if the two items co-occur alongside each other frequently.

To calculate *tweet affect*, we averaged the affective ratings of the tweets’ component words. Tweets composed entirely of words absent from the Sentiment140 lexicon were excluded from the analysis (these correspond to approximately 0.03% of our dataset). We manually compiled event prediction data for each candidate. Using computational methods, we also compiled twitter data and tweet affect for each candidate. We then matched the two datasets based on the candidate names. This matching was done computationally, using Python, prior to our regression analysis and other related statistical tests.

We were especially interested in tweets posted after 6am EST on November 5^th^, after election results had been announced. We chose 6am as it provided a single point of time at which all election results in our analysis had been announced–we do not expect our result to change with minor modifications to the cutoff time, as most of the tweets were created later on in the morning of November 5^th^). There were 101,283 such tweets. Candidate-level variability in post-election tweet affect might be confounded with pre-election expectations; candidates who were expected to win might have systematically higher or lower tweet ratings than candidates expected to lose. We thus standardized affective ratings for tweets after 6am on November 5^th^ by subtracting the average pre-election affective ratings of tweets for each candidate before 6am on November 4^th^ (before Election Day). This adjustment gives us the *change in tweet affect* pre and post-election–with a positive score implying that post-election tweet affect was more positive than average pre-election tweet affect for that candidate. The average change in tweet affect after 6am EST on November 5th was -0.003 (*SD* = 0.21, min = -1.70, max = 2.55), indicating that tweet affect dropped slightly after election results were announced. Summary statistics for the change in tweet affect, and other relevant variable, are provided in S1 File.

### Results

To determine whether change in tweet affect depended on expectations about the candidate’s chances of winning, we created binary scores for less certain and more certain beliefs referred to as weak and strong expectations, respectively. The expected outcome scale ranged from -4 to +4, and our cutoffs were +/- 2 points (corresponding to an expectation of “safe” or “very safe” for the candidate or their opponent). The analysis compared changes in tweet affect for winners who were strongly expected to win (defined as expected outcome ≥ 2) with winners who were weakly expected to win (2 > expected outcome). We also compared changes in tweet affect for losers who were weakly expected to lose (expected outcome > -2) with losers who were strongly expected to lose (-2 ≥ expected outcome). These four categories corresponded to expected wins, surprising wins, surprising losses, and expected losses.

Expectations were far from random; there was a strong correlation between the candidate’s expected outcome and the percentage of votes that was obtained (*r =* 0.85, *p* < 0.001). A binary form of the expected outcome (greater or less than zero) perfectly predicted the election outcomes in all cases except North Carolina.

**Fig 1:**
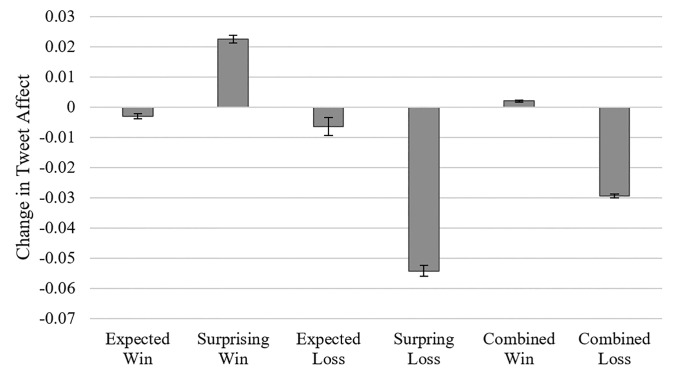
Average change in tweet affect for candidates who won and were strongly or weakly expected to win (*expected win* and s*urprising win*), and candidates who lost and were strongly or weakly expected to lose (*expected loss* and *surprising loss*) in Study 1. This figure also displays change in tweet affect for all winners and all losers (*Combined Win* and *Combined Loss*). Error bars correspond to +/- 1 standard error.

As shown in Fig 1, winning candidates who were just weakly expected to win had a more positive changes in tweet affect than those who were strongly expected to win (*β* = 0.025, *t* = 16.29, 95% CI = [0.022, 0.029], *Cohen’s d* = 0.12, *p* < 0.001) indicating that surprising wins generated more positive affect than expected wins. This test and all other tests, unless explicitly mentioned, were conducted on the level of individual tweet rather than aggregate data. Likewise, losing candidates who were just weakly expected to lose had a more negative changes in tweet affect than those who were strongly expected to lose (*β* = -0.047, *t* = -14.15, 95% CI = [-0.055, -0.041], *Cohen’s d* = -0.22, *p* < 0.001), indicating that surprising losses generated more negative affect than expected losses. There was also a large difference in the change in tweet affect for candidates who won and candidates who lost, as expected (*β* = 0.044, *t* = 24.90, 95% CI = [0.041, 0.047], *Cohen’s d* = 0.15, *p* < 0.001). Note that these results do not change if we use a cutoff value of +/-2.6, corresponding to the median absolute expectation, rather than +/- 2 points.

The above analysis provides a convenient overview of the key trends in our data, however it does not accommodate candidate-level characteristics such as party affiliation and incumbency. It also excludes variability in the data by considering only binary measures of expectation (strong vs. weak expectation). Thus, we more rigorously examined the relationship between change in tweet affect (dependent variable) and expectation about the elections’ outcomes (independent variable) in linear regressions. Our regressions controlled for the outcome (whether the candidate won or lost) as well as the candidate’s party affiliation and incumbency. Consistent with Fig 1, there was a negative effect of expected outcome (*β* = -0.0065, *t* = -6.69, 95% CI = [-0.0085, -0.0047], *p* < 0.001), indicating that stronger expectations were associated with more negative changes in tweet affect, controlling for final outcomes (winning or losing). The more expected the outcome, the more negative the change in affect. There was also a positive effect of winning (*β* = 0.052, *t* = 16.45, 95% CI = [0.046, 0.588], *p* < 0.001). Finally, Democrats had more negative tweet affect than Republicans (*p* < 0.001), but there were no differences in affect depending on incumbency (*p* = 0.42).

As shown in Fig 1, negative surprises have more influence on emotions than positive surprises. To examine this, we conducted a second regression predicting change in tweet affect, with the above variables, as well as an interaction between winning and the expected outcome. The interaction revealed the influence of the expected outcome on tweet affect for winning candidates relative to losing candidates. The coefficient for the expected outcome was negative (*β* = -0.010, *t* = -6.02, 95% CI = [-0.012, -0.006], *p* < 0.001), and the interaction between this variable and the winning variable was positive (*β* = 0.004, *t* = 2.21, 95% CI = [0.000, 0.007], *p* < 0.05). Stronger expectations had a negative effect on affect when candidates won (*β* = -0.010 + 0.004 = -0.006) and lost (*β* = -0.010), and the effect was stronger for losers than winners.

Lastly, we examined whether the results held if the outcome of the election was defined as the percentage of votes the candidate received, rather than the binary variable indicating whether a candidate won or lost. Thus we replicated the above analysis, but replaced the binary variable with the percentage of votes received. Expectations still influenced change in tweet affect (*β* = -0.012, *t* = 11.16, 95% CI = [-0.014, -0.010], *p* < 0.001), and there was a positive effect of vote proportion on change in tweet affect (*β* = 0.010, *t* = 21.13, 95% CI = [0.009, 0.011], *p* < 0.001). A one point increase in vote percentage had roughly the same effect on change in tweet affect as a one point decrease in expectations (on a scale of -4 to 4). The detailed outputs of all the regressions performed above are presented in S1 File.

In order to test the robustness of our findings, we ran the above regressions with absolute tweet affect as our dependent variable, and expected outcome as our independent variable. The controls included not only election outcomes and candidate characteristics, but also average tweet affect for the candidate prior to 6am EST on November 4th (that is, prior to the start of Election Day). This change did not alter our findings, and the effect of expectations on absolute tweet affect was negative and significant in all variants of the above regressions (*p* < 0.001 for all).

Taken together, these results demonstrate that the intensity of affective responses varies as a function of people’s beliefs about uncertain outcomes, consistent with models that presuppose expectation-based affective response. However, a limitation of Study 1 is that expected outcomes were measured on a 9-point scale and therefore didn’t match the scale of actual outcomes which were percentages of votes obtained by each candidate. It was impossible to determine a precise expected vote percentage to compare to actual vote percentages. Our dataset also contained only one election per candidate, so we could not disentangle candidate-level effects from expectation effects. Study 2 addresses these issues.

## Study 2: American Football games

### Materials and methods

This study examined tweets about American Football games played in the 2014/2015 regular and post season of the National Football League (NFL). Just like in the case of Senate elections, results of individual games are highly uncertain and are widely discussed on social media such as Twitter. In addition, there are large betting markets for NFL games, the most common being point spreads. These are predictions about relative points scored by the teams in a game. Point spreads indicate which team is more likely to win and by how many points. Importantly, point spreads and outcomes of the games are on the same scale. Teams that win with a margin greater than the point spread or lose with a margin smaller than the point spread can be seen as exceeding expectations. Teams that win with a margin smaller than the point spread or lose with a margin greater than the point spread can be regarded as falling short of expectations. We obtained point spreads immediately prior to the start of games for the 2014/2015 NFL regular and post season. We used five different forecasters (Betonline.ag, 5Dimes.eu, SportsBetting.ag, BOVADA.lv, and Fantasy911.com), whose forecasts were conveniently summarized on the ESPN.com website. We obtained average point spreads immediately prior to the start of games, and recoded the point spread into an *expected score difference* variable that captured the expected net gain or loss in points relative to the opponent team. For example, an expected score difference of 5 in favor of a team indicated that that team was expected to win with a margin of 5 points. Expected and final score differences for each team in each game were correlated (*r =* 0.40, *p* < 0.001). Expected score differences did not predict all of the outcomes; in 31% of the games, the team predicted to win (i.e. the one with a positive expected score difference) ultimately lost.

Once again we used Twitter API data streaming to download NFL related tweets. We considered only tweets that mentioned one of the 32 teams in the NFL in a *hashtag* format (e.g. #Patriots, #Packers etc.) and streamed these tweets continuously from September 3, 2014 (a day before the start of the regular season) to February 2, 2015 (a day after the end of the season). We only used tweets created less than 12 hours before the start of each game and those created less than 12 hours after the end of each game. We did this to maximize the likelihood that the tweets involving the teams were about the game in consideration (rather than another event or topic related to the team). Data on start and end times of each game were obtained from www.pro-football-reference.com. We also only used tweets that mentioned a single NFL team, and we generated an affective rating for each tweet using the Sentiment140 lexicon, discarding tweets composed of words not in the lexicon (roughly 0.02% of all tweets). Due to an electrical outage, we were unable to download tweets on October 12, 2014, making it impossible to study the 13 games played on this day. However, we obtained tweets before, during, and after every other game, giving us 7,515,023 tweets, for 254 different games between September 2014 and February 2015. The tweet data was ‘mixed’ with the event prediction data using the same methods as in Study 1. Again note that our study is purely observational, in compliance with Twitter user agreements, and the tweets were analyzed without any threat to personal privacy.

Again, we were primarily interested in tweets created in the 12 hour period after each game (total 2,026,794 tweets in our dataset). We standardized affective ratings for these tweets by subtracting the average affective rating of tweets for that same team created in the 12 hour period before the game. In this way, we measured the *change in tweet affect* after the game relative to before the game, for each team in each game. The average change in tweet affect was -0.033 (*SD* = 0.27, min = -5.24, max = 4.98). Again, this type of standardization is useful to control for confounds associated with team and game-level variability (e.g., teams expected to win a game could have more enthusiastic supporters and a higher absolute tweet affect). Summary statistics for the change in tweet affect, and other relevant variables, are provided in S1 File.

### Results

To examine associations among tweet affect, expectations, and actual outcomes, we divided the data based on the median absolute expected score difference, which was 4.4.

**Fig 2:**
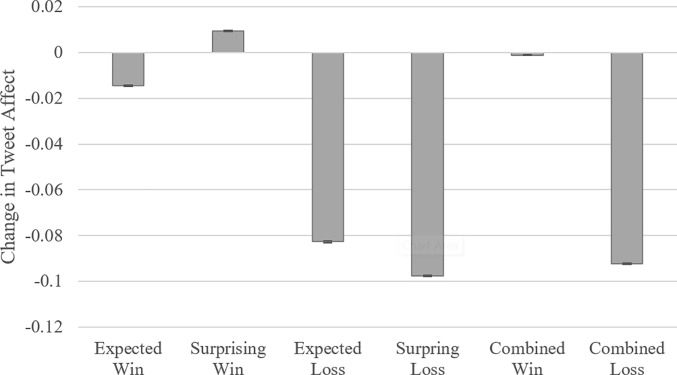
Average change in tweet affect for teams that won and were strongly or weakly expected to win (*expected win* and s*urprising win*), and teams that lost and were strongly or weakly expected to lose (*expected loss* and *surprising loss*) in Study 2. This figure also displays change in tweet affect for all winners and all losers (*Combined Win* and *Combined Loss*). Error bars correspond to +/- 1 standard error.

Comparisons involved winning teams that were weakly vs. strongly expected to win (*expected score difference* < 4.4 and *expected score difference* ≥ 4.4, respectively) and losing teams that were weakly vs. strongly expected to lose (*expected score difference* > -4.4 and *expected score difference* ≤ -4.4, respectively). Average changes in tweet affect for these four groups appear in Fig 2. Losing teams that were weakly expected to lose had a more negative change in tweet affect than losing teams that were strongly expected to lose (*β* = -0.015, *t* = -22.57, 95% CI = [-0.016, -0.013], *Cohen’s d* = -0.06, *p* < 0.001), and winning teams that were weakly expected to win had a more positive change in tweet affect than winning teams that were strongly expected to win (*β* = 0.024, *t* = 51.06, 95% CI = [0.0.23, 0.025], *Cohen’s d* = 0.09, *p* < 0.001). Additionally, as shown in Fig 2, winners had a more positive change in tweet affect than losers (*β* = 0.091, *t* = 231.00, 95% CI = [0.090, 0.092], *Cohen’s d* = 0.34, *p* < 0.001). Once again, surprising losers were associated with more negative affect than expected losers, and surprising winners were associated with more positive affect than expected winners.

The above analysis describes the key trends in our data, however it does not accommodate game-level characteristics such as whether the game was played at the team’s home stadium and whether the game was in the regular or the post season. It also excludes variability in the data by considering only binary measures of expectation (strong vs. weak expectation). We thus conducted a linear regression on our entire dataset, with change in tweet affect as the dependent variable and the (continuous) expected score difference for the associated team as the independent variable. This analysis controlled for game outcome (winning or losing), whether the game was played at the team’s home stadium, and whether the game was in the regular or the post season. With multiple games per team, we also included team-level random intercepts. There was a negative effect of expected score difference (*β* = -0.0044, *t* = -96.67, 95% CI = [-0.0045, -0.0043], *p* < 0.001) and a positive effect of winning (*β* = 0.106, *t* = 243.90, 95% CI = [0.106, 0.107], *p* < 0.001), indicating that stronger expectations were associated with lower affect, controlling for games’ final outcomes.

We also tested the robustness of the results to different measures of the outcome of the game. We ran a regression using final score difference, rather than the binary game outcome (winning or losing), as the control variable. This regression also yielded a negative effect of expected score difference (*β* = -0.0029, *t* = -6.85, 95% CI = [-0.0029, -0.0028], *p* < 0.001) and a positive effect of final score difference (*β* = 0.0022, *t* = 147.84, 95% CI = [0.0022, 0.0022], *p* < 0.001). The relative size of the *β* coefficients indicates that a one point decrease in the expected score difference for a team had a slightly larger effect on tweet affect than a one point increase in the final score difference. Home teams and post season games were associated with tweets that expressed more positive affect (*p* < 0.001). Detailed outputs of the above regressions are provided in S1 File.

Again, it is useful to see whether the results hold when team and game-level controls are implemented in a slightly different manner. We used absolute tweet affect as our dependent variable, and a control variable capturing the average affective rating of tweets for the team created in the 12 hour period before the game. This change did not alter the findings (*p* < 0.001 for all effects discussed above).

With this dataset, we could transform the outcome of the game, i.e. the final score difference, into a variable that captured the difference between the final and the expected score. This *relative final score difference* tells us how much better a team performed compared to pre-game expectations. When this variable was positive, the team either won with a greater margin than expected or lost with a lower margin than expected. Likewise, if this variable was negative, the team either lost with a greater margin than expected or won with a lower margin than expected.

In order to rigorously test the effect of surpassing or falling below expectations, we examined changes in tweet affect as a function of relative final score difference. This was done using a discontinuity regression. We created a binary variable that measured whether the team exceeded expectations or not and a second variable that measured the interaction between exceeding the expectation and the relative final score difference. These variables were included with the relative final score difference and team and game-level controls to predict changes in tweet affect. The relative final score difference had a positive effect (*β* = 0.0038, *t* = 66.05, 95% CI = [0.0037, 0.0039], *p* < 0.001) indicating that higher final scores relative to expectations were associated with more positive tweets. We also found a positive effect of exceeding expectations (*β* = 0.020, *t* = 33.01, 95% CI = [0.019, 0.021], *p* < 0.001), indicating that there was a significant jump in tweet affect as teams surpassed expectations. This jump implies that tweet affect changed in a discrete manner for teams exceeding vs. falling below expectations. Lastly, there was a negative interaction between exceeding expectations and relative final score difference (*β* = -0.0027, *t* = -58.69, 95% CI = [-0.0028, -0.0026], *p* < 0.001). The effect of expectations was weaker when expectations were surpassed than when expectations fell short. The detailed output of this regression is provided in S1 File.

**Fig 3:**
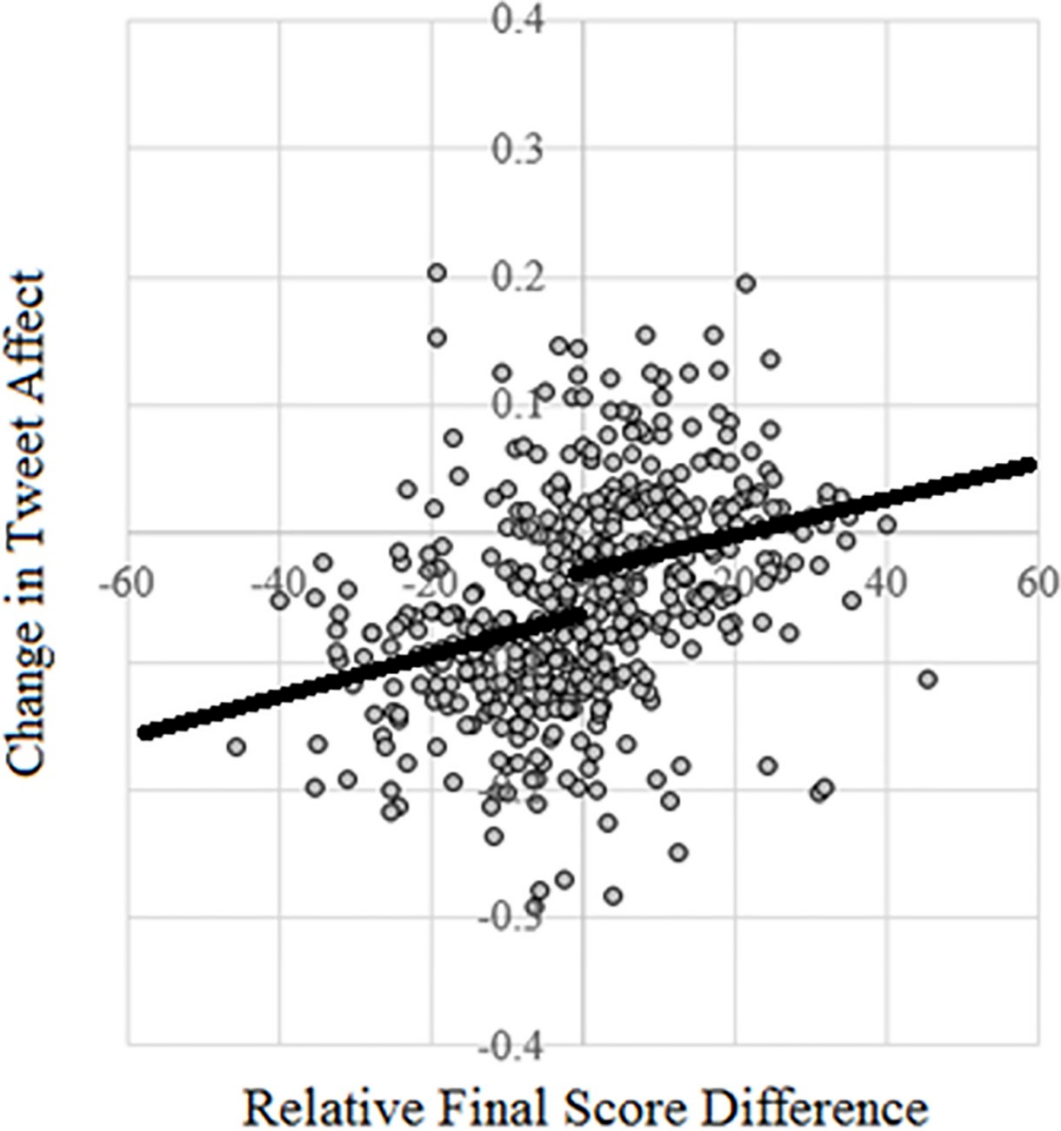
The average change in tweet affect for each team in each game plotted against the relative final score difference for the team in the game, in Study 2. The solid lines show predictions of the discontinuity regression performed on pooled data. As indicated by this regression, there is a discrete jump in change in tweet affect as teams surpass expectations.

Our results can be best understood by visually examining the average change in tweet affect for each team in each game and comparing it with the relative final score difference associated with a given team for a given game. Fig 3 displays a scatter plot of the pooled data. There is a positive association between change in tweet affect and relative final score difference. Teams that scored many more points than expected received more positive tweet affect than those that scored fewer points than expected. Teams that performed just as well as expected did not display strong changes in tweet affect. A linear regression testing this relationship on the pooled data in Fig 3 showed a positive association (*β* = 0.002, *t* = 8.63, 95% CI = [0.0016, 0.0026], *p* < 0.001).

We also examined whether the relationship between change in tweet affect and relative final score difference varied when the team surpassed or fell short of expectations. The dependent variable was the average change in tweet affect, and independent variables were whether the team exceeded or fell short of expectations, the relative final score difference, and the interaction between them. There was a positive effect of relative final score difference (*β* = 0.0015, *t* = 2.70, 95% CI = [0.0004, 0.0025], *p* < 0.01) and a positive effect of exceeding expectations (*β* = 0.025, *t* = 2.27, 95% CI = [0.0034, 0473], *p* < 0.05), indicating a discrete jump in tweet affect as teams surpassed expectations. Finally, there was a negative interaction between exceeding expectations and relative final score difference, however the interaction was not statistically significant (*p* = 0.88), perhaps due to the small sample size of the pooled data (*N* = 508).

Overall, the intensity of affective responses depends on expectations, with outcomes greatly exceeding or falling short of expectations having the strongest responses. Consistent with the results of Study 1, we also find that expectations also influence the size of the asymmetry between affective responses to gains and losses.

## Discussion

Data from platforms such as Twitter (which is now one of the primary forums for personal and public discourse) provide valuable opportunities to explore a wide range of psychological topics, many of which are otherwise difficult to study in the lab. Recent work has used Twitter data to screen populations for psychological risk factors [18], evaluate theories of collective behavior [19], create automated assessment of personality [20], examine temporal orientation [21], explore perceptions of time [22], investigate the determinants of wellbeing [23], and study of the effects of inequality on brand preferences [24]. Targeted interventions on twitter have been also showed to reduce the use of racial slurs on this social platform [25]. In fact, a recent systematic review found 382 publications that used Twitter data between 2006 and 2012 across various disciplines [26]. A more recent review found 137 publications that utilized Twitter data to study health-related topics alone [27]. With the advancement of natural language processing methods, Twitter data are being used to predict real world outcomes in the domains of finance, politics, public health, and crime [28]. Although much of this work uses information about sentiment to generate predictions, this research can be further improved by drawing on established theories in the psychological sciences.

Our work expands on important findings from two large-scale naturalistic studies of the affective responses to political and sporting events. Using historical datasets of football matches and lottery gambling, Otto et al. [29] showed that more lottery tickets are purchased following an unexpected rather than expected win for the home team. The authors suggested that this malleability of risk attitudes is driven by the positive mood associated with a surprising, positive event. In a similar vein, Healy et al. [30] assessed the relationship between electoral performance of the incumbent party for US Senate elections (1964–2008) and the results of the college football games that took place in the same county as the election and prior to the day of elections. The authors showed that victory of the local team was associated with an increased support for the incumbent’s party vote share (by 0.81 percentage point when the team won a day before the elections). Using point spreads from the betting market to quantify expectations that people held about the games’ outcomes, the authors showed that surprising successes of the local teams are associated with more positive evaluations of the incumbent party (i.e. status quo). Healy et al. [30] further corroborated these findings in a survey conducted during the 2009 NCAA college basketball tournament. Participants were interviewed at different points during the tournament, being asked about their support for the current president (Barack Obama). In addition, half of the participants were reminded about the basketball performance of their favorite team prior to providing their political opinions. Those who were reminded about the football results expressed increased support for the president if their favorite team won and that outcome was surprising. Healy and colleagues attributed these effects to the increase in wellbeing caused by the surprising successes of the favorite teams.

Both studies show how expectations and emotions contribute to decisions in a naturalistic setting. However, neither of these studies measures the affective changes associated with the results of expected and unexpected events. In the present work, we used the sentiment of people’s online communication to quantify this affective change, rigorously demonstrating the interplay of expectation and affect.

The increased availability of *big data*, as well as the development of more sophisticated tools for analyzing such data, allow several extensions of this paper. For example, research in computational linguistics has developed techniques that go beyond valence-based assessments of language and discourse. These techniques can automatically assess which emotional states are being expressed in a piece of text, and can subsequently be used to test theories of behavior involving the divergent effects of similarly valenced emotions [31, 32]. Other work has developed tools to obtain demographic data from social media profiles and can be used to better understand social, economic, and geographic variability in affective outcomes [33]. Large-scale online data have also been used to study cognitive processes such as categorization and memory [34]. These efforts help to shed light on the cognitive underpinnings of affective responses in natural environments. Finding novel ways to observe emotion, belief, and behavior, and test psychological theories in the real world is an exciting direction for future work.

## Supporting information

S1 File Summary statistics of variables and outputs of additional regressions. (DOCX)
