# Supplementary material for: Affective responses to uncertain real-world outcomes: Sentiment change on Twitter
Source: PLoS One. 2019 Feb 27;14(2):e0212489. doi: 10.1371/journal.pone.0212489 (PMC6392292; doi:10.1371/journal.pone.0212489)
Supplement: S1 File — (DOCX) [file pone.0212489.s001.docx]

Supporting Information

Table A: Summary statistics for variables

| *Variable* | *Mean* | *SD* | *Min* | *Max* |
| --- | --- | --- | --- | --- |
|  |  |  |  |  |
| *Study 1* |  |  |  |  |
| *change in tweet affect* | -0.0024 | 0.2117 | -1.7028 | 2.5525 |
| *expectation* | 1.4303 | 1.6378 | -3.8000 | 3.8000 |
| *voteshare* | 53.0604 | 3.7107 | 29.5000 | 62.1000 |
| *won* | 0.8305 | 0.3752 | 0.0000 | 1.0000 |
| *incumbent* | 0.5963 | 0.4906 | 0.0000 | 1.0000 |
| *democrat* | 0.1607 | 0.3672 | 0.0000 | 1.0000 |
|  |  |  |  |  |
|  |  |  |  |  |
| *Study 2* |  |  |  |  |
| *change in tweet affect* | -0.0321 | 0.2698 | -5.2444 | 4.9891 |
| *expectation* | 1.2652 | 6.0342 | -14.1000 | 14.1000 |
| *won* | 0.6608 | 0.4735 | 0.0000 | 1.0000 |
| *playoff* | 0.2211 | 0.4150 | 0.0000 | 1.0000 |
| *teamhome* | 0.5488 | 0.4976 | 0.0000 | 1.0000 |
| *teamscorerel* | 4.2385 | 14.4448 | -52.0000 | 52.0000 |

Table B. Study 1: Regression of change in tweet affect on expectation, with controls for election outcome (binary variable corresponding to winning or losing), as well as incumbency and party status.

|  | *Coef* | *SE* | *z* | *p* | *95% CI-L* | *95% CI-H* |
| --- | --- | --- | --- | --- | --- | --- |
| *won* | 0.0540 | 0.0032 | 16.7200 | 0.0000 | 0.0477 | 0.0604 |
| *expectation* | -0.0066 | 0.0010 | -6.6900 | 0.0000 | -0.0085 | -0.0047 |
| *incumbent* | -0.0007 | 0.0023 | -0.2900 | 0.7700 | -0.0051 | 0.0038 |
| *democrat* | -0.0473 | 0.0020 | -23.9300 | 0.0000 | -0.0512 | -0.0434 |
| *constant* | -0.0298 | 0.0026 | -11.6700 | 0.0000 | -0.0349 | -0.0248 |

Table C. Study 1: Regression of change in tweet affect on expectation, with controls for election outcome (continuous variable corresponding to vote share), as well as incumbency and party status

|  | *Coef* | *SE* | *z* | *p* | *95% CI-L* | *95% CI-H* |
| --- | --- | --- | --- | --- | --- | --- |
| *voteshare* | 0.0099 | 0.0005 | 21.1300 | 0.0000 | 0.0090 | 0.0109 |
| *expectation* | -0.0117 | 0.0010 | -11.1600 | 0.0000 | -0.0137 | -0.0096 |
| *incumbent* | -0.0219 | 0.0020 | -11.0500 | 0.0000 | -0.0258 | -0.0181 |
| *democrat* | -0.0163 | 0.0028 | -5.9200 | 0.0000 | -0.0217 | -0.0109 |
| *constant* | -0.4978 | 0.0240 | -20.7300 | 0.0000 | -0.5449 | -0.4508 |

Table D. Study 1: Regression of change in tweet affect on expectation, with controls for election outcome (binary variable corresponding to winning or losing), as well as incumbency and party status, and interaction between winning and expectation.

|  | *Coef* | *SE* | *z* | *p* | *95% CI-L* | *95% CI-H* |
| --- | --- | --- | --- | --- | --- | --- |
| *won* | 0.0558 | 0.0033 | 16.7600 | 0.0000 | 0.0493 | 0.0623 |
| *expectation* | -0.0091 | 0.0015 | -6.0200 | 0.0000 | -0.0121 | -0.0062 |
| *incumbent* | -0.0019 | 0.0023 | -0.8000 | 0.4230 | -0.0064 | 0.0027 |
| *democrat* | -0.0469 | 0.0020 | -23.5800 | 0.0000 | -0.0508 | -0.0430 |
| *interaction* | 0.0035 | 0.0016 | 2.2100 | 0.0270 | 0.0004 | 0.0067 |
| *constant* | -0.0328 | 0.0029 | -11.3600 | 0.0000 | -0.0385 | -0.0272 |

Table E. Study 2: Regression of change in tweet affect on expectation, with controls for game outcome (binary variable corresponding to winning or losing), as well as whether the game was played at home and whether it was a playoff game. Random effects applied on team level.

|  | *Coef* | *SE* | *z* | *p* | *95% CI-L* | *95% CI-H* |
| --- | --- | --- | --- | --- | --- | --- |
| *expectation* | -0.0044 | 0.0000 | -96.6700 | 0.0000 | -0.0045 | -0.0043 |
| *won* | 0.1064 | 0.0004 | 243.9000 | 0.0000 | 0.1055 | 0.1072 |
| *teamhome* | 0.0252 | 0.0005 | 55.4300 | 0.0000 | 0.0243 | 0.0261 |
| *playoff* | 0.0045 | 0.0006 | 7.6600 | 0.0000 | 0.0034 | 0.0057 |
| *constant* | -0.1206 | 0.0055 | -21.8300 | 0.0000 | -0.1314 | -0.1098 |

Table F. Study 2: Regression of change in tweet affect on expectation, with controls for game outcome (continuous variable corresponding to relative score), as well as whether the game was played at home and whether it was a playoff game. Random effects applied on team level.

|  | *Coef* | *SE* | *z* | *p* | *95% CI-L* | *95% CI-H* |
| --- | --- | --- | --- | --- | --- | --- |
| *expectation* | -0.0029 | 0.0000 | -63.8500 | 0.0000 | -0.0030 | -0.0028 |
| *teamscorerel* | 0.0022 | 0.0000 | 147.8400 | 0.0000 | 0.0022 | 0.0022 |
| *teamhome* | 0.0233 | 0.0005 | 50.5600 | 0.0000 | 0.0224 | 0.0242 |
| *playoff* | 0.0068 | 0.0006 | 11.2500 | 0.0000 | 0.0056 | 0.0080 |
| *constant* | -0.0612 | 0.0055 | -11.0900 | 0.0000 | -0.0721 | -0.0504 |

Table G. Study 2: Regression of change in tweet affect on relative final score difference, exceeding expectations, interaction between relative final score difference and exceeding expectations, with controls for game outcome (continuous variable corresponding to relative score), as well as whether the game was played at home and whether it was a playoff game. Random effects applied on team level.

|  | *Coef* | *SE* | *z* | *p* | *95% CI-L* | *95% CI-H* |
| --- | --- | --- | --- | --- | --- | --- |
| *exceedexpec* | 0.0202 | 0.0006 | 33.0100 | 0.0000 | 0.0190 | 0.0214 |
| *relfinscorediff* | 0.0038 | 0.0001 | 66.0500 | 0.0000 | 0.0037 | 0.0039 |
| *interaction* | -0.0027 | 0.0000 | -58.6900 | 0.0000 | -0.0028 | -0.0026 |
| *teamscorerel* | -0.0006 | 0.0000 | -13.5500 | 0.0000 | -0.0007 | -0.0005 |
| *teamhome* | 0.0268 | 0.0005 | 57.5900 | 0.0000 | 0.0259 | 0.0277 |
| *playoff* | -0.0025 | 0.0006 | -3.9700 | 0.0000 | -0.0037 | -0.0013 |
| *constant* | -0.0575 | 0.0057 | -10.1300 | 0.0000 | -0.0686 | -0.0464 |
